# Effect of *Aloe barbadensis* Mill. formulation on Letrozole induced polycystic ovarian syndrome rat model

**DOI:** 10.4103/0975-9476.74090

**Published:** 2010

**Authors:** Radha Maharjan, Padamnabhi S. Nagar, Laxmipriya Nampoothiri

**Affiliations:** Department of Biochemistry, M.S. University of Baroda, Vadodara, Gujarat, India.; 1 Department of Biochemistry, M.S. University of Baroda, Vadodara, Gujarat, India.; 2 Department of Botany, M.S. University of Baroda, Vadodara, Gujarat, India.

**Keywords:** Aloe vera, anovulation, infertility, insulin resistance, letrozole, polycystic ovary syndrome.

## Abstract

This is a preliminary study that explores the efficacy of Aloe vera gel formulation as a possible therapeutic agent in the prevention and management of polycystic ovary syndrome (PCOS). PCOS is recognized as the most common endocrinopathy of women. Increased androgen synthesis, disrupted folliculogenesis, and insulin resistance lie at the patho-physiological core of PCOS. Current therapy for such a syndrome is use of insulin sensitizers. Large randomized clinical trials of metformin as the insulin-sensitizing drug, however, suggested that it produces many side effects after prolonged usage. For this reason, an alternate therapy would be to use herbs with hypoglycemic potential. *Aloe barbadensis* Mill. (Liliaceae) popularly known as *Aloe vera* is a well-known plant with such properties. The present study evaluated the efficacy of Aloe vera gel formulation in a PCOS rat model. Five month old Charles Foster female rats were orally fed with letrozole, a non-steroidal aromatase inhibitor, to induce PCOS. The rats were then treated orally with the Aloe vera gel formulation (1 ml dose daily for 45 days). This restored their estrus cyclicity, glucose sensitivity, and steroidogenic activity. Co-treatment of the inductive agent (letrozole) with the Aloe vera gel prevented the development of the PCO phenotype. Aloe vera gel formulation exerts a protective effect in against the PCOS phenotype by restoring the ovarian steroid status, and altering key steroidogenic activity. This can be attributed to phyto-components present in the extract.

## INTRODUCTION

Polycystic ovary syndrome (PCOS) is one of the most common endocrine disorders among women affecting 4-10% of those of reproductive age.[1,2] PCOS is characterized by hyperandrogenism, insulin insensitivity, and chronic anovulation.[3] Research over the last few decades has established that PCOS is an important metabolic disorder, associated with an increased risk of T2DM as well as metabolic syndrome.[4] It has been proposed women who have mild hyperandrogenism and an isolated ultrasonic finding of polycystic ovaries but whose ovulatory function is maintained exhibit a mild form of PCOS. These women may be susceptible to developing the syndrome as well.[5] Increased luteinising hormone (LH) and increased insulin levels mainly amplify the intrinsic abnormality of their steroidogenesis. In PCOS, excess androgen activity may alter gonadotropin-induced estrogen and progesterone synthesis in the follicles.[6] Normally, testosterone and androstenedione are converted to estradiol and estrone, respectively, with help of P450 aromatase, which plays an important role in the ovary’s hormonal balance. However, decreased activity of this enzyme results in the increased ovarian androgen production and development of the PCO condition.[7]

Over the last few decades, research has established that PCOS is a prevalent metabolic disorder associated with T2DM.[8] Current available modes of treatment use insulin sensitizers like metformin since the central core of PCOS etiology is through insulin resistance.[9,10] But these drugs have been reported to have side effects after prolonged usage.[11,12] Hence, researchers today are exploring alternative therapies to treat and manage such infertility problems.[13,15] In this context, the role of certain medicinal plants in the control of hyperglycemic conditions has been demonstrated.[16,17,21] Aloe vera is one such plant, for which hypoglycemic effects have been explored along with several other medical applications.[18,19] For example, an alcoholic extract of Aloe vera gel (AVG) maintained glucose homeostasis of streptozotocin induced diabetes rats by controlling carbohydrate metabolizing enzymes.[20,22] This action has been ascribed to various poly-phenols present in the mixture.[23] In addition, plant sterols identified in Aloe greatheadii possess similar glucose lowering effects.[24] These observations led us to evaluate an AVG formulation as a possible therapeutic agent to manage and prevent PCOS.

## AIM OF STUDY

To evaluate the efficacy of AVG on the letrozole induced polycystic ovarian syndrome (PCOS) rat model.

## MATERIALS AND METHODS

### Authentication procedure

*Aloe barbadensis* Mill. (Voucher no. PSN 723) was compared with the specimen (Bhatt 2486, 653, 279, JVJ 448) at the nationally recognized BARO Herbaria of the Department of Botany, M.S. University of Baroda, Vadodara, Gujarat, India.

### Formulation preparation

Fresh mature Aloe vera leaves (3.5-years old) were taken and washed with water, incised with a sterilized knife and allowed to stand for 2 hr in order to remove the aloin. Later, the gel was removed by separating the epidermis and sonicated to get a homogenous gel. Further, to the prepared gel, turmeric (*Curcuma longa* L. [3--5 g/l]), karaya gum (*Sterculiaurens* Roxb. [8--10% g/l]), and lemon juice (*Citrus limon* L. [1%]) were added as natural preservatives. This was stored at 4°C.

### Phytochemical analysis

Phyto components were studied by preliminary phyto chemical analysis. Polyphenol content of AVG was studied by incubating with 1 ml of Folin ciocalteu’s reagent and 0.8 ml of Na_2_CO_3_ (7.5%) for 90 min and colored complex was measured at 765 nm.[25] Estimation of sterols was done by incubating AVG with Libermann Burchard reagent, which results into a colored complex, determined spectrophotometrically at 640 nm.[26] For flavonoid estimation, AVG was mixed with assay mixture containing 750 μl of 95% of ethanol, 50 μl of aluminum chloride (AlCl_3_), 50 μl of 1M acetic acid, and 1.4 ml of distilled water measured at 415- nm.[27]

### Animals

The PCOS rat model was developed with adult virgin Charles Foster female rats weighing 200--225g. All animals were checked daily for 4 day ovarian cycle using vaginal cytology. All animals were kept under controlled conditions of light and temperature and having free access to diet and water. The rats were divided into two experimental groups: control group of animals (*n*=6--8) received orally 1% aqueous solution of carboxymethylcellulose (CMC) and another group of animals (*n*=6--8) were treated orally with letrozole (0.5 mg/kg body weight) daily for 21 days.[28] Oral Glucose Tolerance Test (OGTT) was performed regularly every 15 day period to check glucose sensitivity. Twenty four hours after their last dose of letrozole, blood was collected to assess toxicological biomarkers. Following this, the animals were sacrificed in the late diestrus stage and their ovaries were removed. One of the ovaries was fixed in Bouins fluid and histological studies were performed. The other ovary was accessed for steroidogenic enzyme activities. All protocols were approved by both the Committee for the purpose of Control and Supervision on Experiments on Animals (CPCSEA) and the University Ethical Committee.

Letrozole treated experimental rats which demonstrated irregular estrus cyclicity, glucose intolerance, and altered steroid status were considered PCOS positive animals and used for further study. These animals were further divided into two groups- PCOS untreated (control) rats and AVG treated rats. The letter were given orally a concentration of 1 ml daily for 45 days (10 mg dry weight of AVG). The other group of animals received both letrozole (inductive agent for PCOS) and AVG together for 45 days daily. All groups were continuously monitored for insulin sensitivity by OGTT. At the end of treatment, the rats were sacrificed and assessed for various biochemical parameters.

### Oral glucose tolerance test

OGTT was performed after 12 hr fasting for all rats in the experiments.[29] Blood samples were collected in fluoride coated anticoagulant vials. Next, glucose (300 mg/kg body weight) was orally fed to the rats and blood samples were collected after time intervals of 30’, 60’, 90’ and120’. The blood was subjected to 3000 rpm for 10 min and the plasma separated. Glucose was estimated using GOD--POD based kits.

### Preparation of ovarian homogenate

10% ovarian homogenate was prepared in 0.1 M Tris HCl buffer (pH--7.8) and centrifuged at 10,000 rpm for 30 min at 4μC. The supernatant was used as a source of steroidogenic enzyme assay, and protein content was monitored.

### Steroidogenic enzyme assays

The key steroidogenic enzymes - 3β hydroxy steroid dehydrogenase and 17β hydroxy steroid dehydrogenase were assayed to evaluate the enzyme activity of ovarian enzyme.[30] In brief, the enzyme assay was carried out in 0.1 M Tris HCl buffer (pH 7.8) containing NAD (500 μM) and the substrate DHEA (100 μM) for 3β hydroxy steroid dehydrogenase or 17β estradiol (100 μM) for 17β hydroxy steroid dehydrogenase in a total volume of 3 ml. The reaction was started by adding the enzyme (100 μl) together with the color reagent, INT. The mixture was then incubated at 37°C for 1 hr. The reaction was terminated by the addition of 2.0 ml of phthalate buffer (pH 3.0) and read at 490- nm. The enzyme activity was calculated from the standard curve for NADH and expressed as n moles of NADH formed per hr per mg protein.

### Histological analysis

Ovaries were removed and fixed in Bouins fixative. Histological examinations of ovaries from all groups were carried out using standardized histological methods. Sections of 5 μm thickness were cut in paraffin-embedded block and stained with hematoxylin eosin.[31]

### *“In vitro”* experiments

To understand the direct effect of AVG on steroidogenic enzymes, we performed “*In vitro*” assay. Ovarian protein (30-35g) was incubated both with and without AVG (1 mg dry weight of aloe gel), for 1 hr at 4°C. In addition to these treatments, ovarian protein was also incubated with metformin [1.2 mg][32] which was used as positive control. The activities of ovarian enzymes 3β HSD and 17β HSD were estimated by the method described by Shivanandappa *et al*., 1997.

### Statistical analysis

Comparison of values was done by One-Way Analysis of Variance (ANOVA) followed by the student t-test. This analysis done by using the Package of Software Graph pad 3.0 version. *P*<0.05 were considered statistically significant.

## RESULTS

The phyto--components present in the AVG formulation were qualitatively (data not shown) and quantitatively analyzed for polyphenols, sterols, flavanoids along with some other nutrients (glucose, protein, cholesterol content)[Table 1].

**Table 1:** Quantitative analysis of phytocomponent of aloe vera gel

Sample AVG	Content
Glucose (g/100 g)	3.2
Protein (mg/g)	1.0
Cholesterol (mg/g)	18.73
Total polyphenols (μg/g)	23.72
Total flavonoid (μg/g)	2.28
Total sterol (μg/g)	65.47

**Figure 1:**
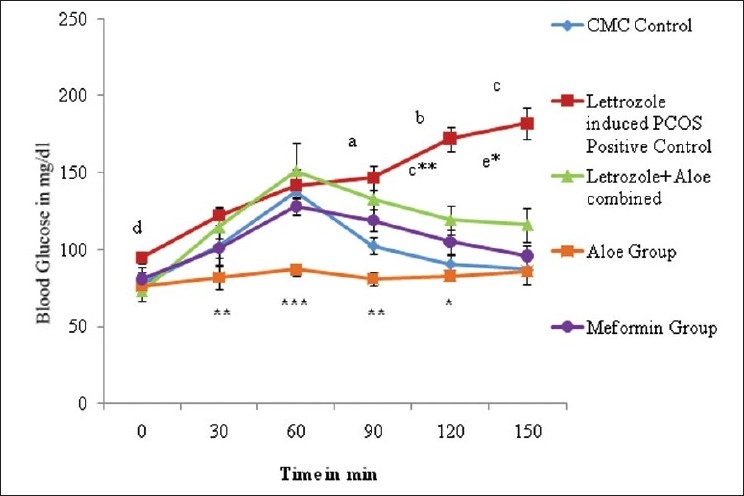
Effect oral administration of aloe vera gel formulation (1 ml/45 days) on oral glucose tolerance test profile of letrozole induced PCOS rats, n=4-6. The values are represented as mean ± SEM a *P*<0.0001, b *P*<0.005, c *P*<0.0002, d *P*<0.01, as compared to control group.***P<0.0001, **P<0.01, *P*<0.05, c**P<0.001, e*P<0.02 as compared to letrozole (PCOS) model.

**Figure 2a:**
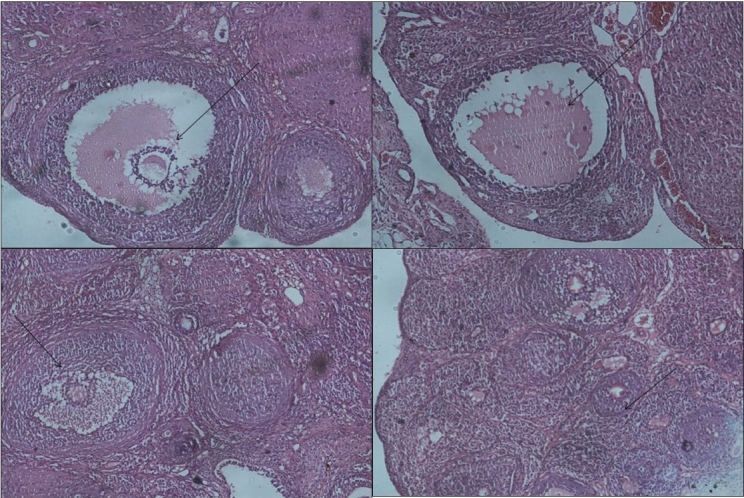
Effect oral administration of aloe vera gel formulation (1 ml/45 days) on follicular growth of ovary in letrozole induced PCOS rats (a) CMC control rat showing normal follicular development (H and E ×10) b: Section of ovary from letrozole treated showing small cysts in the follicle (x10) c: Section of ovary from aloe treated group showing normal follicle growth (x10) d: Section of ovary from metformin treated group showing normal primary follicle growth (x10)

**Figure 3:**
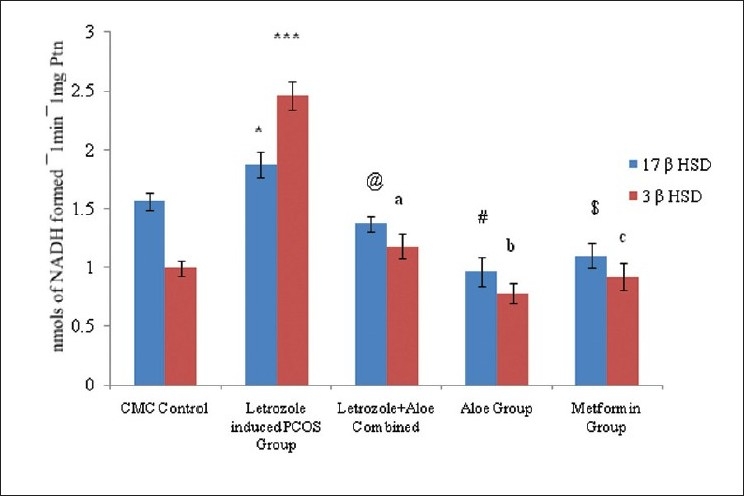
Effect oral administration of aloe vera gel (1 ml /45 days) on steroid dehydrogenase enzyme activity in letrozole induced PCOS rats, n=4-6, The values are represented as mean ± SEM, *P<0.05, ***P<0.001, as compared to Control Group, ^@^P <0.05 ^a^*P*<0.001, ^#^*P*<0.02, ^b^*P*<0.01, ^$^*P*<0.002, ^c^*P*<0.01 as compared to PCOS Group

Rats treated with letrozole for induction of PCOS showed significant increases in body weight and ovarian weight, and altered estrus cyclicity compared to controls. PCOS positive animals exhibited an increase in glucose intolerance compared to controls. [Figure 1]. PCOS rats exhibited many small atretic cysts [Figure 2b], whereas no histological abnormalities were observed in control rats [Figure 2a]. Key ovarian steroidogenic enzymes 3β hydroxysteroid dehydrogenase and 17β hydroxysteroid dehydrogenase showed increases in activity in letrozole induced PCOS rats compared to control rats. [Figure 3]

Letrozole induced PCO untreated rats exhibited a significant increase *P*<0.004 in body weight compared to controls. In contrast, no further increase in body weight was observed in AVG treated PCO rats. AVG treatment in letrozole induced PCO rats also demonstrated showed similar kinds of rhythm in estrus cyclicity as controls and the metformin group suggesting reversion toward normal physiology Letrozole treated PCO rats showed significant increases in ovarian weights (*P*<0.001) whereas AVG treated PCO animals exhibited ovarian weights similar to controls. The OGTT profile showed the letrozole induced PCOS group to have significant *P*<0.0001 [Figure 1] glucose intolerance compared to the control and metformin groups whereas the combined treatment (letrozole and AVG treated simultaneously) as well as AVG treated PCO animals exhibited an improvement in glucose sensitivity. Histological analysis exhibited a decrease in ovary atretic cysts after AVG treatment of PCO rats [Figure 2c] compared to PCO controls [Figure 2b] and normal growth similar to [Figure 2a] metformin controls [Figure 2d]. As represented in Figure 3, AVG treatment in letrozole induced PCO animals caused an improvement in ovarian 3β hydroxy steroid dehydrogenase (3β HSD) and 17β hydroxy steroid dehydrogenase (17β HSD) activities, comparable to both control and metformin treated rats suggesting improvement in steroid status. AVG treatment in PCO rats showed no effects on biomarker enzymes indicating that treatment does not affect major organ systems, namely kidney and liver function[33,34] [Table 2].

**Table 2:** Effect of AVG (1 ml orally / 45 days) on biomarkers of toxicity letrozole induced PCOS rats

Groups	Control	Let (PCOS)	Let+aloe combined	Aloe group	Metformin group
Serum Glutamate Pyruvate Transaminase (SGPT) mg/dl	59.33±2.12	59.2±1.08	46.25±1.23	59.4±2.05	61±1.28
Creatinine mg/dl	0.59±0.25	0.57±0.17	0.50±0.31	0.58±0.11	0.62±0.21

N=3-4. The values are mean + SEM

**Figure 4:**
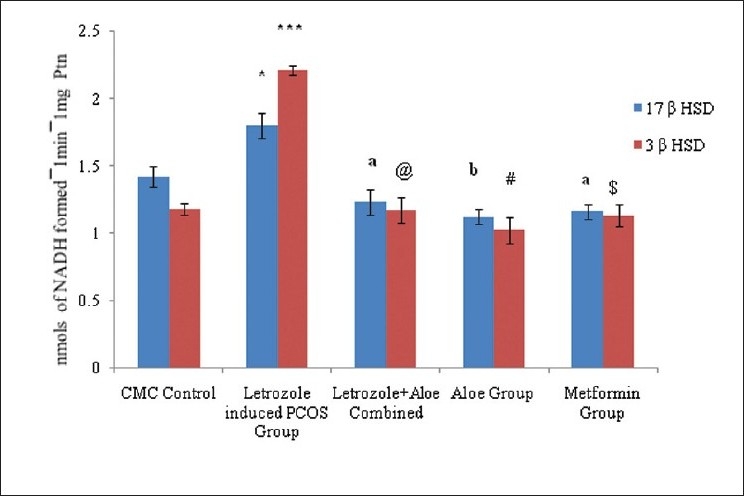
*“In vitro”* effect of aloe vera gel formulation on ovarian steroidogenic enzyme activity on letrozole induced rats model, n=4-6. The values are represented as mean ± SEM, *P<0.001, ***P<0.003 as compared to Control Group, ^a^*P* <0.05, ^@^*P*<0.02, ^b^*P*<0.004, ^#^*P*<0.003, ^$^*P*<0.002 as compared to PCOS Group.

Direct effect of AVG was evaluated by *in vitro* incubation with PCOS rat ovarian protein. Ovarian steroidogenic enzymes 3β HSD and 17β HSD activity in *“in vitro”* incubations yielded activity similar to control and metformin groups, suggesting that AVG acts directly on the ovarian enzymes [Figure 4].

## DISCUSSION

PCOS has many clinical manifestations, including oligomenorrhea and hyper-androgenism, leading to metabolic dysfunction.[35] In the present study, we investigated the biochemical and clinical characters of PCOS in a rat model. The inducing drug inhibited aromatase, thereby increasing ovarian androgens, leading to hyperandrogenism, a hallmark of PCOS.[36] Similarly, we found a significant weight gain in letrozole treated PCO compared to control rats which was attributable to deposition of abdominal fat.[37,38]

PCOS is positively correlated with insulin resistance. The PCOS rat model was examined for glucose intolerance, finding that these rats exhibited hyperglycemic tendencies contributing to insulin resistance, leading to full hyperglycemia and metabolic syndrome.[39] Thus, insulin resistance may be a consequence of increased truncal fat and high levels of free fatty acids.

Similarly, the present study found the AVG-treated PCOS rats to have returned to normo glycemic condition from their hyperglycemic condition. This may be attributable to nutritionally rich phytosterols and phyto-phenols present in the plant.[18,19]

In PCOS, excess production of androgens interferes with the process of follicular maturation and selection of dominant follicles during ova formation. It also promotes early stages of follicular growth in primate ovary leading to the syndrome’s insulin resistance and fat distribution. In our study, PCO rats demonstrated the formation of empty cysts filled with follicular fluid similar to reported ovarian histology. In all these ways, the rat model behaves similarly to the human system indicating that it adequately mimics the human PC ovary.

In the model, hyperinsulinemia is also positively correlated with estrogen deficiency[40,41] as in PCOS. As estrogen synthesis is inhibited by the use of inhibitor in our model, the 3β HSD activity is higher compared to 17β HSD activity, and androgen production will be higher than estrogen production. This will affect LH: FSH hormonal balance. Thus we can state that AVG treatment brought 3β HSD activity in PCO rats back to normal levels comparable to those in the controls.

Reversion of estrus cyclicity to normal following AVG treatment could be attributed to phytochemical components present in the gel that maintain steroid status, enabling fertility status to be regained.

Preliminary phytochemical data found AVG rich in phytosterols and polyphenols that could be active components in controlling hyperglycemic conditions and modulating steroidogenes. However, no reports of phytosterols or polyphenols affecting steroidogenic enzymes have been published. We hypothesize that these phyto--components act at various stages of the steroidogenic enzyme cascade and modulate the activity back to normal. Data from our *“in vitro”* study indicate that AVG acts directly on key enzymes like 3β HSD, decreasing enzyme activity and modulating the flux toward estradiol formation. However, the specific phyto--component acting on the enzyme system needs to be identified.

In conclusion, the present study indicates that AVG has potential efficacy in the prevention and maintenance of PCOS.
